# The Loss of the p53 Activator HIPK2 Is Responsible for Galectin-3 Overexpression in Well Differentiated Thyroid Carcinomas

**DOI:** 10.1371/journal.pone.0020665

**Published:** 2011-06-17

**Authors:** Luca Lavra, Cinzia Rinaldo, Alessandra Ulivieri, Emidio Luciani, Paolo Fidanza, Laura Giacomelli, Carlo Bellotti, Alberto Ricci, Maria Trovato, Silvia Soddu, Armando Bartolazzi, Salvatore Sciacchitano

**Affiliations:** 1 Research Center, St. Pietro Fatebenefratelli Hospital, Rome, Italy; 2 Department of Experimental Oncology, Molecular Oncogenesis Laboratory, Regina Elena Cancer Institute, Rome, Italy; 3 Department of Pathology, St. Andrea Universitary Hospital, Rome, Italy; 4 Department of Surgical Sciences, University of Rome “Sapienza”, Rome, Italy; 5 Chair of Surgery, University of Rome “Sapienza”, St. Andrea Hospital, Rome, Italy; 6 Departement of Clinical and Molecular Medicine, University of Rome “Sapienza”, Rome, Italy; 7 Department of Human Pathology, University of Messina, Policlinico “G Martino Universitary Hospital, Messina, Italy; 8 Cellular and Molecular Tumour Pathology Laboratory, Cancer Center Karolinska, Karolinska Hospital, Stockholm, Sweden; Emory University, United States of America

## Abstract

**Background:**

Galectin-3 (Gal-3) is an anti-apoptotic molecule involved in thyroid cells transformation. It is specifically overexpressed in thyroid tumour cells and is currently used as a preoperative diagnostic marker of thyroid malignancy. Gal-3 expression is downregulated by wt-p53 at the transcriptional level. In well-differentiated thyroid carcinomas (WDTCs) there is an unexplained paradoxical concomitant expression of Gal-3 and wt-p53. HIPK2 is a co-regulator of different transcription factors, and modulates basic cellular processes mainly through the activation of wt-p53. Since we demonstrated that HIPK2 is involved in p53-mediated Gal-3 downregulation, we asked whether HIPK2 deficiency might be responsible for such paradoxical Gal-3 overexpression in WDTC.

**Methodology/Principal Findings:**

We analyzed HIPK2 protein and mRNA levels, as well as loss of heterozygosity (LOH) at the *HIPK2* locus (7q32-34), in thyroid tissue samples. HIPK2 protein levels were high in all follicular hyperplasias (FHs) analyzed. Conversely, HIPK2 was undetectable in 91.7% of papillary thyroid carcinomas (PTCs) and in 60.0% of follicular thyroid carcinomas (FTCs). *HIPK2* mRNA levels were upregulated in FH compared to normal thyroid tissue (NTT), while PTC showed mean *HIPK2* mRNA levels lower than FH and, in 61.5% of cases, also lower than NTT. We found LOH at *HIPK-2* gene locus in 37.5% of PTCs, 14.3% of FTCs and 18.2% of follicular adenomas. To causally link these data with Gal-3 upregulation, we performed *in vitro* experiments, using the PTC-derived K1 cells, in which HIPK2 expression was manipulated by RNA interference (RNAi) or plasmid-mediated overexpression. HIPK2 RNAi was associated with Gal-3 upregulation, while HIPK2 overexpression with Gal-3 downregulation.

**Conclusions/Significance:**

Our results indicate that HIPK2 expression and function are impaired in WDTCs, in particular in PTCs, and that this event explains Gal-3 overexpression typically observed in these types of tumours. Therefore, *HIPK2* can be considered as a new tumour suppressor gene for thyroid cancers.

## Introduction

The family of the *Homeodomain Interacting Protein Kinase* (*HIPK*) genes was discovered thirteen years ago. Their ability to interact and phosphorylate specific serine/threonine residues in many different targets and partners has been extensively studied. By virtue of their protein/protein interaction, HIPKs are involved in the regulation of gene transcription and in cell response to DNA damage [1]. HIPK2, the most studied member of the family, acts as co-regulator of an increasing number of transcription factors and modulates many different basic cellular processes such as apoptosis, proliferation, DNA damage response, differentiation, and development. Most of these effects are mediated by phosphorylation and activation of the oncosuppressor protein p53 [2]–[5]. However, the exact role of HIPK2 in the development and progression of human cancer is not clear yet. Recently, two missense mutations of the *HIPK2* gene have been identified in acute myeloid leukaemia (AML) and in myelodysplastic syndrome (MDS), a pre-leukaemia syndrome [6]. However, extensive search failed to detect any mutation in many other tumours. An alternative mechanism of HIPK2 inactivation, described in breast cancer, is its cytoplasmic relocalization mediated by the interaction with HMGA1 that causes inhibition of nuclear activation of wt-p53 apoptotic function [7]. Experiments performed in normal rat thyroid epithelial PC Cl3 cells demonstrated that HIPK2 exerts a potent inhibitory effect on cell growth, and this effect is mediated by its kinase activity [8]. In another preliminary study, *HIPK2* gene expression was analyzed in a panel of 14 thyroid carcinomas and a 3 to 10-fold reduction in its mRNA expression levels was observed in 8 of them [9]. Recently, we demonstrated that HIPK2 is involved in the p53-mediated repression of the anti-apoptotic factor Galectin-3 (Gal-3) [10]. Gal-3 is a β-galactoside-specific lectin with anti-apoptotic activity, involved in both tumourigenesis and resistance to chemotherapeutic drugs [11]–[12]. Gal-3 posses the functional BH1 domain of the Bcl-2 family [13], inhibits cytochrome-c release from mitochondria [14], and is aberrantly expressed in different types of human cancers [15]. *In vitro* experiments demonstrated that Gal-3 expression is required for the maintenance of the transformed phenotype of papillary thyroid carcinoma (PTC)-derived cells [16] and is responsible for chemoresistance and refractoriness to conventional treatments of PTCs [17]. I*n vivo* studies, performed on well differentiated (WDTCs) and anaplastic thyroid tumours (ATCs), demonstrated that Gal-3 expression was restricted to the cytoplasm of malignant thyroid follicular cells [18], [19]. Based on the results of a prospective multicenter study, Gal-3 overexpression is now considered as a sensitive marker of thyroid malignancy and it is currently used in the preoperative diagnosis of thyroid cancer [20]. In our previous study, we demonstrated that, in poorly differentiated thyroid carcinomas (PDTCs) and ATCs, the occurrence of a gain-of-function p53 mutation not only leads to the loss of the capability to downregulate Gal-3 but it acquires a *de novo* ability to stimulate its expression and induce chemoresistance [19]. However, in WDTCs there is an unexplained paradoxical concomitant expression of Gal-3 and wt-p53 [21], [22]. Since HIPK2 is involved in p53-mediated Gal-3 downregulation, we asked whether HIPK2 deficiency might be responsible for such paradoxical behaviour. In the present study we show that: i) HIPK2 protein expression is lost in PTCs; ii) *HIPK2* mRNA levels are up-regulated in follicular hyperplasia (FH) and reduced in PTC and in follicular variants of PTC (FVPTC); iii) loss of heterozygosity (LOH) affecting the *HIPK2* gene locus can be detected in more than one third of PTCs; iv) RNA interference (RNAi) or overexpression of HIPK2 in PTC-derived K1 cells causes upregulation or downregulation respectively of Gal-3 expression.

Taken together, our results show the loss of the p53 activator HIPK2 in WDTC and the concomitant upregulation of the anti-apoptotic factor Gal-3. These results may explain the paradoxical co-expression of wt-p53 and Gal-3 in these types of tumours and suggest that *HIPK2* can be considered as a tumour suppressor gene in thyroid cancers.

## Results

### HIPK2, p53 and Gal-3 protein expression analysis

In order to analyze HIPK2 protein levels in wt-p53-carriyng WDTCs and to correlate it with Gal-3 protein expression, a total of 43 thyroid lesions from patients of group A, including 14 FHs, 24 PTCs and 5 follicular thyroid carcinomas (FTCs) (Table 1), have been analyzed by immunohistochemistry (IHC) for the expression of HIPK2, p53 and Gal-3 (Table 2).

**Table 1:** Clinicopathological features of group A patients analyzed by IHC.

A			
case #	age	sex	diagnosis
1	38	F	FH
2	67	M	FH
3	46	M	FH
4	36	F	FH
5	70	F	FH
6	51	F	FH
7	62	F	FH
8	51	F	FH
9	50	F	FH
10	24	F	FH
11	32	F	FH
12	63	M	FH
13	49	F	FH
14	47	M	FH

B						
case #	age	sex	diagnosis	TNM	stage	size (cm)
15	77	F	PTC	T3 N1a M0	III	9
16	23	F	PTC	T1b N1 M0	I	2
17	41	F	PTC	T3 N1 M0	I	3.2
18	23	F	PTC	T3 N0 M0	I	2.5
19	35	F	PTC	T1a N0 M0	I	1
20	27	F	PTC	T3 N1b M0	I	1
21	20	F	PTC	T1b N1 M0	I	1.8
22	46	M	PTC	T3 N0 M0	III	1.5
23	30	M	PTC	T3 N1b M0	I	4.2
24	56	M	PTC	T1a N0 M0	I	1
25	26	F	PTC	T3 N1b M0	I	2.5
26	79	M	PTC	T3 N0 M0	III	5
27	46	F	PTC onco.	T1b N0 M0	I	1.7
28	48	F	PTC onco.	T1a N0 M0	I	0.9
29	32	F	PTC onco.	T1b N0 M0	I	1.5
30	40	F	FVPTC	T3 N0 M0	I	4
31	59	F	FVPTC	T1a N0 M0	I	1
32	60	F	FVPTC	T3 N0 M0	III	1.5
33	42	F	FVPTC	T3 N0 M0	I	1.2
34	64	F	FVPTC	T1a N0 M0	I	1
35	75	F	FVPTC	T1a N0 M0	I	1
36	73	M	FVPTC	T1a N0 M0	I	2
37	66	F	FVPTC	T3 N1b M0	IV A	3.8
38	36	F	FVPTC	T1b N0 M0	I	1.2
39	55	F	FTC	T1b N0 M0	I	2
40	28	M	FTC	T3 N0 M0	I	4.5
41	21	F	FTC	T2 N0 M0	I	3.4
42	63	F	FTC onco.	T2 N0 M0	II	3.3
43	81	F	FTC onco.	T3 N0 M0	III	8

Patients were divided, according to histological diagnosis, in FHs (A) and WDTCs (B).

**Table 2:** HIPK2, p53 and Gal-3 protein expression analysis in thyroid tumours by IHC.

Case #	HIPK2	p53	Gal-3
*Follicular Hyperplasia (FH)*			
1	+	Neg.	Neg.
2	+	Neg.	Neg.
3	+	Neg.	Neg.
4	+	Neg.	Neg.
5	+	Neg.	Neg.
6	+/−	Neg.	Neg.
7	+	Neg.	Neg.
8	+	Neg.	Neg.
9	+	Neg.	Neg.
10	+	Neg.	Neg.
11	+	Neg.	Neg.
12	+	Neg.	Neg.
13	+	Neg.	Neg.
14	+	Neg.	Neg.
*Papillary Thyroid Carcinoma (PTC)*			
15	Neg.	Neg.	++
16	Neg.	Neg.	++
17	+/−	Neg.	++
18	Neg.	Neg.	++
19	Neg.	Neg.	++
20	Neg.	Neg.	++
21	+/−	Neg.	++
22	Neg.	Neg.	++
23	Neg.	Neg.	++
24	Neg.	Neg.	++
25	Neg.	Neg.	++
26	Neg.	Neg.	++
*Oncocytic PTC*			
27	Neg.	Neg.	Neg.
28	Neg.	Neg.	++
29	Neg.	Neg.	++
*Follicular Variant of PTC (FVPTC)*			
30	Neg.	Neg.	+
31	Neg.	Neg.	++
32	Neg.	Neg.	++
33	Neg.	Neg.	++
34	Neg.	Neg.	++
35	Neg.	Neg.	++
36	Neg.	Neg.	+/−
37	Neg.	Neg.	++
38	Neg.	Neg.	++
*Follicular Thyroid Carcinoma (FTC)*			
39	Neg.	Neg.	++
40	Neg.	Neg.	+/−
41	Neg.	Neg.	Neg.
*Oncocytic FTC*			
42	+/−	Neg.	++
43	+	Neg.	+

Expression analysis of HIPK2, p53 and Gal-3 in thyroid lesions of group A with the indicated origin, using biotin-free IHC. Results are expressed as: Neg when no immunostaining was observed, +/− when immunostaining was restricted to less than 10% of malignant cells, + when positivity was seen in 10%–70% of cells, and ++ when more than 70% of malignant cells was positive to immunostaining.

**Figure 1:**
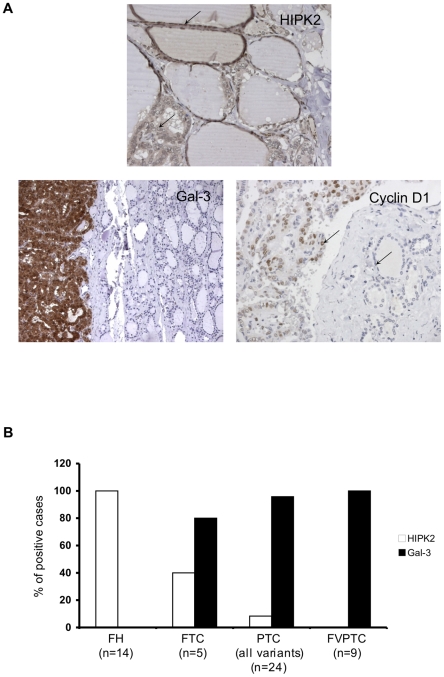
Inverse correlation between HIPK2 and Gal-3 expression in WDTCs. (A) Representative immunohistochemical analysis of HIPK2, Gal-3 and Cyclin D1 in PTC. Arrows indicate positive immunostaining for HIPK2 (upper panel) and Cyclin D1 (lower, right panel) into the nuclear compartment of respectively normal and papillary thyroid cells. In the lower left panel is clearly visible cytoplasmic expression of Gal-3 in tumoural tissue. (B) The histogram shows, for each histotype, the percentage of positive cases at the immunohistochemical expression analysis for HIPK2 (white bars) and Gal-3 (black bars).

HIPK2 protein expression analysis revealed a nuclear immunostaining in all FHs analyzed (Table 2; Figure 1, panels A, B). Conversely, HIPK2 was absent in almost all PTC samples; in particular, 22 out of 24 PTCs analyzed (91.7%) were negative for HIPK2 immunostaining and the remaining 2 cases (8.3%) showed expression of HIPK2 in less than 10% of cells (Table 2; Figure 1, panel B). It is noteworthy that all oncocytic PTC and all FVPTC samples were negative for HIPK2 protein expression (Table 2; Figure 1, panel B). Moreover, 3 out of 5 (60.0%) FTCs analyzed were negative for HIPK2 expression and the remaining two cases (40.0%) showed HIPK2 immunostaining (Table 2; Figure 1, panel B).

To test if the nuclear clearing observed in PTCs could alter the immunoreactivity of the nuclear compartment in these specific tumours, we analyzed the expression of Cyclin D1 by using IHC [23]. As shown in Figure 1, Cyclin D1 nuclear expression was sharply detectable in the clear nuclei of PTC cells (Figure 1, panel A), supporting the fact that lack of HIPK2 immunoreactivity observed in PTCs is a specific event and it is not due to an artefact.

P53 protein expression was negative in all thyroid samples analyzed (Table 2). Immunohistochemical p53 negativity is generally considered indicative of the absence of p53 mutations [24]. This result suggests that all thyroid lesions analyzed express a wt-p53, in agreement with previous studies that reported the occurrence of p53 mutation only in poorly differentiated or in undifferentiated thyroid carcinomas [21]–[22].

Immunohistochemical analysis confirmed the lack of Gal-3 expression in all FHs examined, in agreement with the previous published results [17] (Table 2; Figure 1, panel A). Among WDTCs, 23 out of 24 PCT (95.8%) and 4 out of 5 FTC (80%) samples expressed high Gal-3 protein levels (Table 2; Figure 1, panel B).

Our results indicate the existence of a strong, inverse correlation between HIPK2 and Gal-3 protein levels. In particular, all FH samples were positive for HIPK2 and negative for Gal-3 staining, while most of WDTCs (79.3%) showed overexpression of Gal-3, associated with the absence of HIPK2 protein expression (Table 2; Figure 1, panel B). This inverse correlation was even stronger in FVPTCs, where 9 out of 9 cases (100%) were Gal-3 positive and HIPK2 negative (Table 2; Figure 1, panel B).

These results show that HIPK2 protein expression is lost in WDTCs and in particular in PTCs and in FVPTCs. Moreover, the inverse correlation between HIPK2 and Gal-3 protein levels suggests that the overexpression of Gal-3 in WDTCs might be related to the absence of HIPK2.

### HIPK2 gene expression analysis

**Figure 2:**
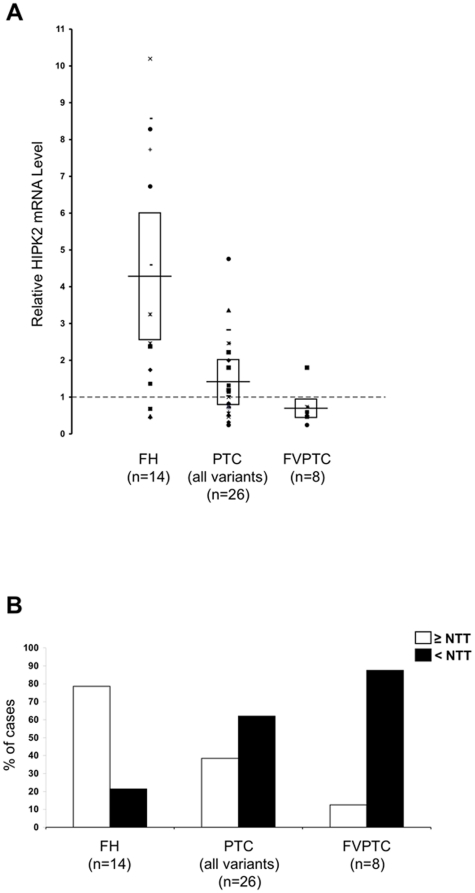
HIPK2 mRNA expression analysis in WDTCs. (A) Real Time RT-PCR analysis of *HIPK2* gene expression in 48 surgical samples of group B patients. Mean *HIPK2* mRNA levels ± SD are shown for FHs, PTCs, and FVPTCs. The results, calculated with 2^ΔΔCT^ method, are expressed relatively to the value of 1.0 obtained analyzing a pool of 10 mRNA extracted from NTT. Horizontal bars indicate the average score within each group of samples, while boxes indicate ±SD. (B) The histogram shows the percentage of samples, within each indicated histotype, with *HIPK2* mRNA expression levels higher (white bars) or lower (black bars) compared to that obtained in the pool of NTT.

To understand the molecular mechanisms responsible for HIPK2 protein expression loss in PTCs, we analyzed *HIPK2* mRNA levels by TaqMan quantitative RT-PCR in a total of 40 thyroid lesions from patients of group B, including 14 FHs and 26 PTCs. The expression of each sample has been calculated relatively to that observed in a pool of 10 normal thyroid tissues (NTT). When compared to the value of 1.0 of the normal pool, FHs showed a mean *HIPK2* expression level of 4.2 (±3.4) (Figure 2, panel A). In PTCs, *HIPK2* mRNA levels were significantly lower than in FH (P<0.005), with a mean level of 1.4 (±1.2) (Figure 2, panel A). Considering only FVPTC samples, *HIPK2* mRNA levels were lower than normal pool with a mean of 0.7 (±0.5) (Figure 2, panel A).

The analysis of the percentage of cases with *HIPK2* expression levels higher or lower than NTT samples demonstrated that in FH, 11 out of 14 cases (78.6%) had *HIPK2* mRNA levels higher than NTT (Figure 2, panel B). Conversely, 16 out of 26 PTCs (61.5%) and 7 out of 8 FVPTCs (87.5%) showed *HIPK2* mRNA levels lower than NTT pool (Figure 2, panel B).

These results demonstrate that *HIPK2* mRNA levels are upregulated in FH with respect to NTT. However, PTC showed mean *HIPK2* mRNA expression levels three times lower than FH and, in the majority of cases, also lower than NTT.

### LOH analysis at the HIPK2 gene locus 7q32-34

**Figure 3:**
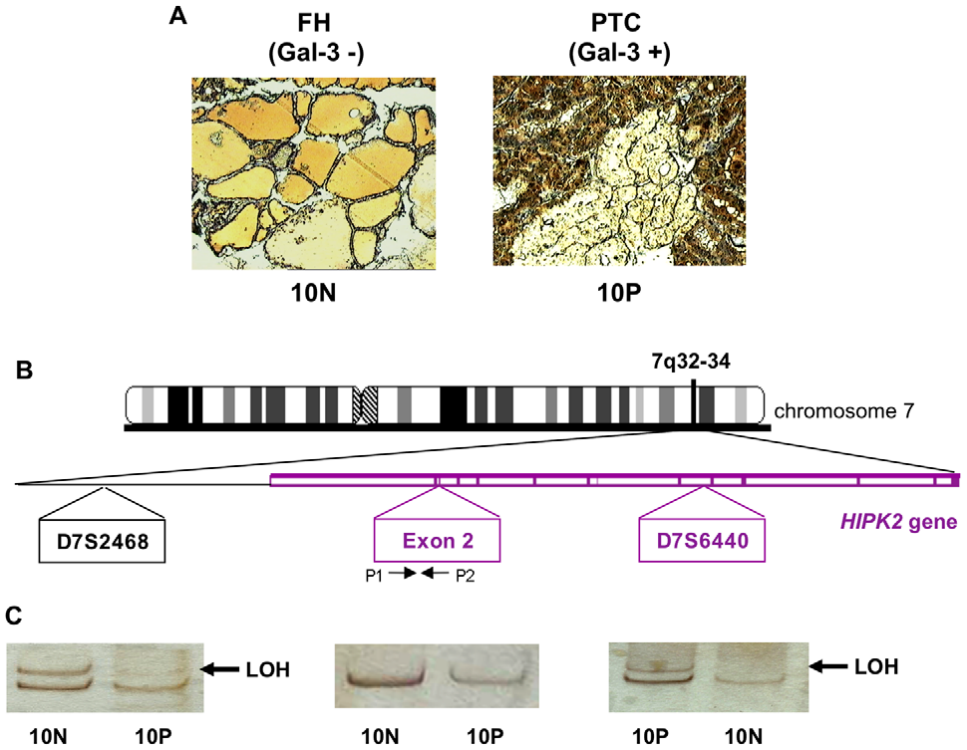
LOH analysis of *HIPK2* gene locus 7q32-34 in WDTCs. (A) Histological pictures of PTC and its matching extra-tumour thyroid tissue immunostained for Gal-3. The upper right panel shows the histological area subjected to laser-based microdissection. (B) Schematic representation of chromosome 7, *HIPK2* locus, and regions including the two microsatellite markers and one *HIPK2* exon amplified by PCR in cells dissected as in (A). The microsatellite amplification products (left and right panels) were resolved onto 12% PAGE and visualized by silver staining. The arrows indicate the missing bands diagnostic of LOH. Quantitative PCR products amplified with the indicated exon 2 primers were resolved and stained as above (central panel).

**Figure 4:**
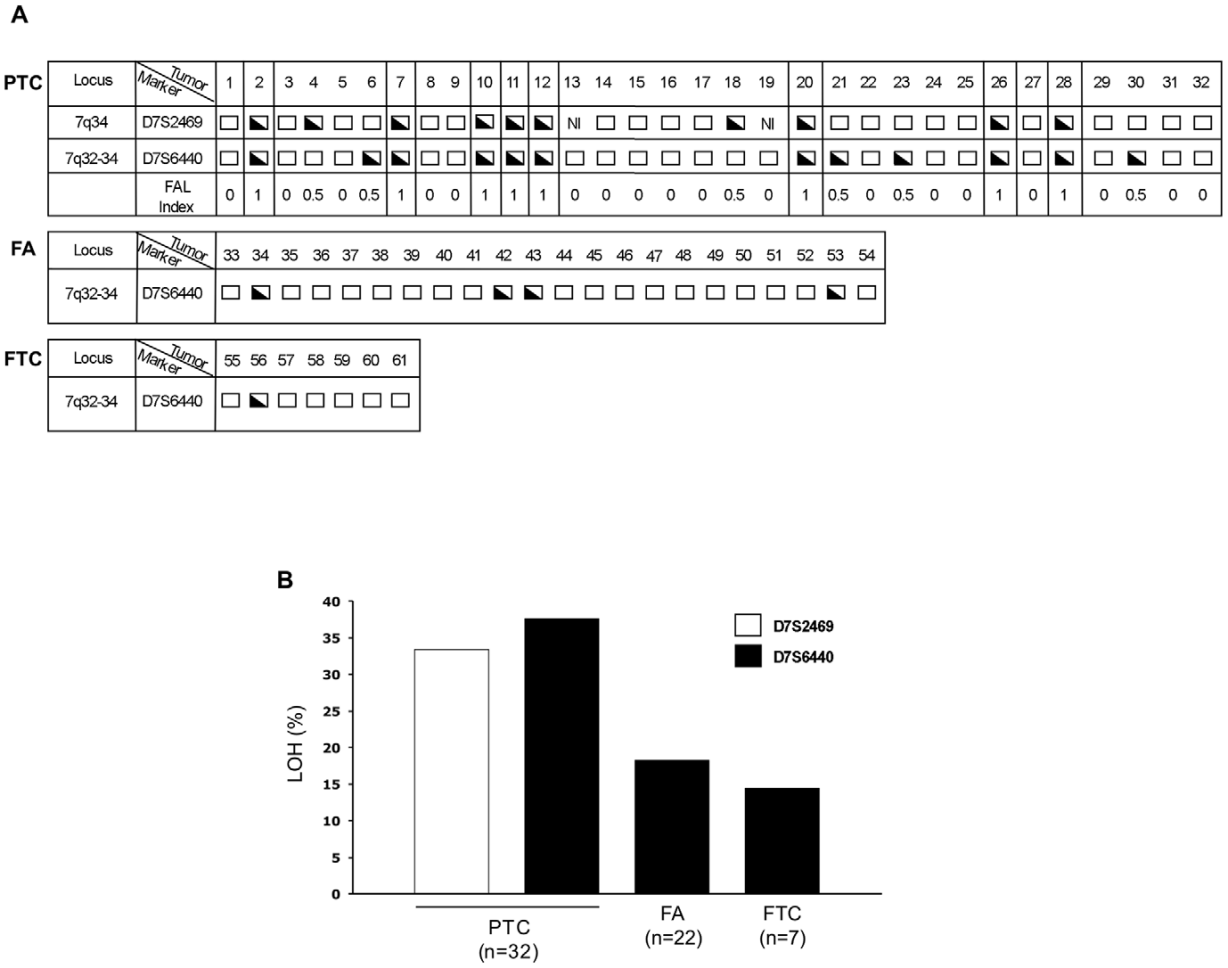
Analysis of allelic loss affecting *HIPK2* gene in WDTCs. (A) Results of LOH analysis at the indicated loci and microsatellite markers in 32 PTCs, 22 FAs and 7 FTCs of group C patients. For each case the retention of heterozigosity (empty box), the LOH (half-filled box), the non-informative condition (NI) as well as the FAL index are reported. PTC cases showing LOH at both microsatellites are highlighted in boxes. (B) The histogram shows the percentage of LOH in each indicated histotype, evaluated at the two microsatellites, namely D7S2469 (white bar) and D7S6440 (black bars).

Allelic loss at the long arm of chromosome 7, where the *HIPK2* gene is located, is a frequent event in PTC [25]–[27]. Thus, we tested whether one of the mechanisms of loss of HIPK2 expression observed in WDTCs might be the loss of genetic material at the *HIPK2* gene locus. We performed LOH analysis at 7q32-34 in 61 patients from group C, including 32 PTCs, 22 follicular adenomas (FAs) and 7 FTCs. Gal-3 expressing tumour cells and matching extra-tumour thyroid follicular cells were selectively isolated by Laser Capture Microdissection (LCM) (Figure 3, panel A). LOH analyses were performed at a microsatellite marker (D7S2468), proximal to the *HIPK2* gene (Figure 3, panels B, C) and at a new microsatellite marker (D7S6440) internal to the *HIPK2* gene that we identified in a non-coding region of intron 9 (Figure 3, panel B). We found LOH in 10 out of 30 informative cases (33.3%) at the proximal marker, and in 12 out of 32 informative cases (37.5%) at the internal marker (Figure 4, panels A, B). LOH at both microsatellite markers was detected in 8 cases and the frequency of allelic loss was higher in the HIPK2-internal microsatellite, indicating that the *HIPK2* gene is located inside the smallest common deleted region. We found LOH for the microsatellite marker D7S6440 in 4 out of 22 (18.2%) FAs and in 1 out of 7 (14.3%) FTC cases (Figure 4, panels A, B). As further control, a portion of the coding region (exon 2) of the *HIPK2* gene was amplified on DNA extracted from microdissected cells. Densitometric analyses of the relative bands showed 50% intensity reduction only in patients with LOH (Figure 3, central panel C) indicating the occurrence of a deletion including at least part of the *HIPK2* gene.

Thus, loss of one copy of the HIPK2 gene is a relatively common event in PTC that may partially contribute to the decrease of HIPK2 mRNA and protein expression levels observed in PTCs.

### Effects of modulation of HIPK2 protein levels on Gal-3 expression

**Figure 5:**
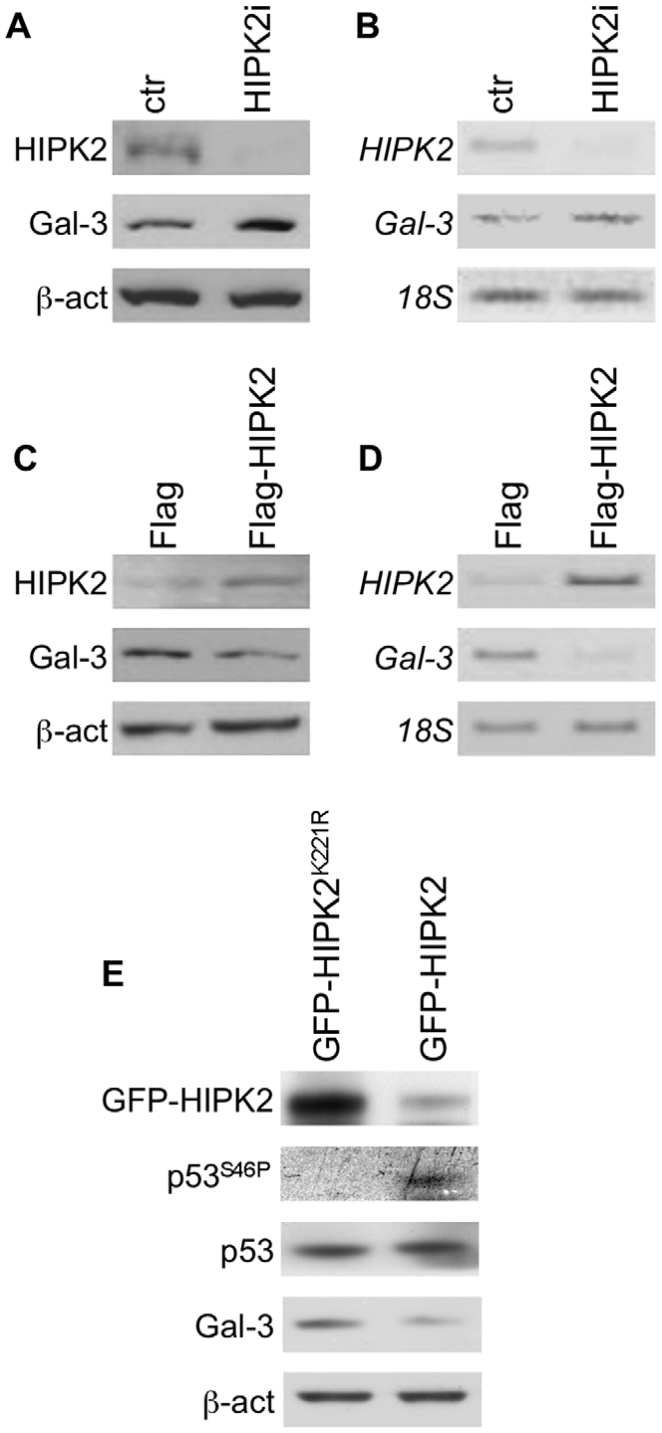
Effects of modulation of HIPK2 protein levels on Gal-3 expression. (A) Western blot analysis, using antibodies to HIPK2 and Gal-3, performed on TCE obtained from PTC-derived K1 cell line stably transfected with pSUPER.retro control vectors (ctr) and pSUPER-HIPK2 interfering construct (HIPK2i). (B) Inverted images of agarose gels showing semiquantitative RT-PCR analyses of *HIPK2* and *Gal-3* gene expression performed on total RNA extracted from K1 cells described in (A). (C) Western blot analysis of HIPK2 and Gal-3 in K1 cell transiently transfected with control vector pCMV-FLAG (Flag) and with pCMV-FLAG-HIPK2 (Flag-HIPK2) expression construct. (D) Inverted images of agarose gels showing semiquantitative RT-PCR analyses of *HIPK2* and *Gal-3* gene expression performed on total RNA extracted from K1 cells described in (C). (E) Western blot analysis of HIPK2, p53Ser46, total p53 and Gal-3 in K1 cell transiently transfected with the kinase-dead pEGFP-HIPK2^K221R^ (GFP-HIPK2^K221R^) and the wild type pEGFP-HIPK2 (GFP-HIPK2) HIPK2 expression constructs. Western blot experiments were normalized using β-actin protein expression while the *18S* gene was amplified as control for semiquantitative RT-PCR analyses.

To demonstrate that Gal-3 overexpression in WDTCs is a consequence of HIPK2 protein loss, we modulated HIPK2 expression levels *in vitro* by RNAi or by vector-mediated overexpression in a wt-p53-carring PTC-derived cell line, namely the K1 cells (ECACC, Salisbury, United Kingdom). Stable RNAi of HIPK2 expression in K1 cells induced upregulation of Gal-3 both at the protein (Figure 5, panel A) and mRNA (Figure 5, panel B) levels. Conversely, overexpression of HIPK2 in the same cells caused a concomitant downregulation of Gal-3 protein (Figure 5, panel C) and mRNA (Figure 5, panel D) levels. Moreover, overexpression of the HIPK2 kinase-dead mutant K221R, in K1 cells was unable to phosphorylate p53 on Ser46 and to downregulate Gal-3 expression levels compared to wt-HIPK2 (Figure 5, panel E). These results demonstrate that Gal-3 protein levels are regulated by HIPK2 expression and kinase activity probably through the specific phosphorylation of p53 protein at its residue Ser46. These data reinforce our hypothesis that the loss of HIPK2 expression observed in WDTC could be responsible for the concomitant overexpression of Gal-3.

## Discussion

Ionizing radiation has been known for a number of years to be associated with an increased risk of developing a thyroid carcinoma. Exposure to radiation, either from the environment or as a result of medical treatments, in particular when radiation is applied to the head and neck region, represents the most common cause of thyroid cancer [28]–[30]. The reason why thyroid cancer is so sensitive to the effects of radiations is not known. The apoptotic pathway triggered by DNA damage is a relevant major target in thyroid cancer tumourigenesis. We recently identified some of the major components of this pathway. In particular, we demonstrated that DNA damage induced by UV irradiation is responsible for the activation of HIPK2 and that this event, in turn, stimulates the phosphorylation of specific serine/threonine residues in p53 protein. Finally, phosphorylated p53 protein induces downregulation of the potent anti-apoptotic molecule Gal-3, by repressing its expression directly at the promoter level. The decrease in Gal-3 mRNA and protein levels facilitates the occurrence of apoptosis [10]. We then demonstrated that in highly aggressive thyroid tumours, characterized by the occurrence of p53 mutations, this pathway is disregulated. UV-induced damage in cells bearing a mutated p53, in fact, not only is no longer able to repress Gal-3, but it exerts a stimulatory effect on Gal-3 expression. Mutant p53-induced Gal-3 overexpression may explain the aggressive phenotype and chemoresistance, typically encountered in ATCs or PDTCs [19]. Therefore, we proposed a model in which the thyroid cells, exposed to radiations, activate the apoptotic pathway HIPK2/wtp53/Gal-3, which physiologically regulates the fate of damaged cells. However, when a gain-of-function p53 mutation occurs, HIPK2/mutp53/Gal-3 axis is no longer protective against the development of cancer and becomes responsible for the acquisition of new additional tumorigenic properties. The weak and unexplained point of this model relies in the paradoxical behaviour of wt-p53 and Gal-3 in WDTCs. It is well documented that such tumours express a wt-p53 and, in the present study, we found undetectable immunohistochemical levels of p53, generally considered as a surrogate of the absence of p53 mutations [24]. In agreement with our model, the presence of a wt-p53 should exert its repressive effect on Gal-3 expression. On the contrary, Gal-3 is highly expressed in WDTCs and, this feature, is so sensitive and specific that it is used as preoperative diagnostic marker [18], [20]. To explain this paradoxical behaviour we postulate that in WDTC wt-p53 protein is inactive because of a yet-to-be-discovered alteration in the upstream mechanisms responsible for its post-transcriptional modifications (acetylation and/or phosphorylation). Phosphorylation, in fact, is a key event in p53 activation and induction of apoptosis [2]–[3]. We focused our attention on HIPK2, one of the major p53 activators, but we cannot exclude that other p53 activators may be involved as well. In this study we demonstrate the loss of HIPK2 in WDTCs, using three different methods, and by analyzing three independent groups of patients. In particular, we analyzed HIPK2 protein expression by IHC in thyroid histological slides, *HIPK2* mRNA expression by Real Time RT-PCR on total RNA extracted from frozen thyroid tissues samples, and genetic loss at *HIPK2* locus, by LOH analysis in thyroid cancer cells, stained with Gal-3, and retrieved by LCM. We found undetectable or very low levels of HIPK2 protein expression in all tumour samples analyzed. *HIPK2* mRNA was reduced, compared to normal tissue, in 61.5% of PTCs and in 87.5% of FVPTCs. Interestingly, HIPK2 decreased expression at mRNA and protein level was observed in all the cases of FVPTCs, suggesting a relevant role of HIPK2 in this tumour type. In this study we report, for the first time, the occurrence of *HIPK2* mRNA overexpression in almost all FHs analyzed. Mean mRNA levels were more than four times higher compared to NTT. This result is in agreement with previous observation reported in hematopoietic and skeletal muscle cells, where HIPK2 expression was increased upon stimulation of cell proliferation, under physiological conditions [31].

The concordance between HIPK2 loss and Gal-3 overexpression was not observed in only two cases (see Table 2). The occurrence of WDTCs characterized by the absence of Gal-3 expression is rather uncommon and it might be related to the occurrence of a more aggressive phenotype [32]–[36]. We do not know the reason why Gal-3 is not expressed in these lesions and do not have any data so far. We can only speculate that a yet-to-be-discovered specific genetic damage might affect *LGALS3* gene. It is interesting to note that HIPK2 loss appears to be correlated to the presence of malignancy with more sensitivity, compared to Gal-3 overexpression. However, in four cases HIPK2 was detectable, at low level, in the presence of Gal-3 overexpression, indicating that mechanisms that do not imply HIPK2 downregulation, but affect its activity or sub-cellular localization, could be involved in thyroid tumourigenesis [7]. One may hypothesize that the combination of analysis of these two protein markers, namely HIPK2 and Gal-3, would be helpful in ameliorating the preoperative recognition of thyroid cancer.

Finally, allelic loss at the *HIPK2* gene locus was found in 37.5% of PTCs. Therefore, we may speculate that genetic events, leading to loss of one allele at the *HIPK2* gene locus, may account for more than one third of the cases. In this regard, it is relevant to note that the *HIPK2* gene locus is located in a region where the presence of fragile sites has been reported [36]–[38]. It is likely that other events, either at the transcriptional and post-transcriptional or at the translational and post-translational levels may explain why HIPK2 mRNA and protein are lost, in the absence of LOH.

In conclusion, the present study demonstrates the loss of HIPK2 expression in WDTC and indicates that such event may be responsible for lack of p53 activation, thus explaining the paradoxical co-expression of a wt-p53 and overexpressed Gal-3. *HIPK2* may represent a new tumour suppressor gene for these types of cancers and may constitute a new potential promising diagnostic marker and therapeutic target.

## Materials and Methods

### Ethics Statement

This study was conducted according to the principles expressed in the Declaration of Helsinki and was approved by the following Ethics Committees: Ethics Committee of St. Andrea Universitary Hospital, Rome, Italy; Ethics Committee of Policlinico Umberto I Universitary Hospital, Rome, Italy; Ethics and Scientific Committee of Policlinico “G. Martino” Universitary Hospital, Messina. Italy.

All patients provided written informed consent for the collection of samples and subsequent analyses.

### Thyroid tissue specimen collection

Thyroid tissue samples were collected at three different Italian Universitary Hospitals, namely St. Andrea and Policlinico Umberto I, in Rome and Policlinico “G Martino”, in Messina. Tissue samples were obtained at surgery, according to local ethical committee. Histological diagnoses were rendered in agreement with WHO guidelines [39], and according to the recently published new criteria [40]. In this study three different and independent groups of thyroid tissue specimens were analyzed: group A, composed of 14 FHs, 24 PTCs and 5 FTCs, was analyzed by IHC (Table 1); group B, composed of 10 NTTs, 14 FHs and 26 PTCs, was analyzed by Real Time RT-PCR; group C, composed of 32 PTCs, 7 FTCs and 22 FAs, was subjected to LOH analysis performed on cells isolated by means of the LCM.

### Immunohistochemical analysis

Formalin-fixed and paraffin-embedded tissue specimens were used to prepare serial tissue sections for conventional morphologic evaluation and immunophenotypical assay. HRP-conjugated rat mAb to Gal-3 (Galectin-3 thyrotest – Space Import-Export S.r.l. Milan, Italy), mouse mAbs to p53 and Cyclin D1 (Dako), and polyclonal antisera to HIPK2 [31] were used in IHC as previously described [18]. Antigen retrieval microwave treatment of tissues slides in 0.01 mol/l citrate buffer pH 6.0 was applied as required. HRP-conjugated goat anti-mouse or anti-rabbit immunoglobulins (Dako) were used as secondary antisera for p53, cyclin D1 and HIPK2 immunostaining respectively, in indirect immunoperoxidase assay. IHC evaluation was performed independently by two experienced pathologists.

### RNA extraction and semiquantitative RT-PCR analysis

RNA extraction and cDNA synthesis were performed as previously described [10]. PCR amplifications were run using 2 µl of cDNA template with an initial denaturation step of 94°C for 5 min followed by 24–30 cycles at 94°C for 1 min, 55°C for 1 min, 72°C for 1 min, and a final extension cycle at 72°C for 7 min. The *18S* RNA was used to normalize the amount of total RNA present in each reaction. Sequences of the primers specific for both *18S* and *Gal-3* were previously reported [10]. PCR products were visualized on a 2% agarose gel after ethidium bromide staining. Three independent experiments were performed for each sample. The intensities of the bands on gels were measured by densitometry, using the NIH ImageJ software (version 1.32j).

### Real Time RT-PCR analysis

The mRNA expression levels of *HIPK2* were analysed in thyroid samples of the group B. FH and PTC lesions were analysed and compared to a pool obtained from the 10 NTT samples, using the TaqMan Gene Expression Assay No. Hs00179759_m1 (Applied Biosystems, Foster City, CA, USA), according to a previously described methodology [41]. The results were normalized with those obtained amplifying the endogenous control *18S*, assay Hs99999901 s1 (Applied Biosystems). The results were calculated using the 2^−ΔΔCT^ method [42] and the mean expression of the NTT pool as calibrator. Results were statistically evaluated using Student's t-test.

### Immuno-Laser capture microdissection and LOH analysis

Slides obtained from thyroid tissue specimens of group C were immunostained for Gal-3, as previously reported, and counter-stained with haematoxylin and eosin. Thyroid follicular cells were microdissected using the Pix Cell II Laser Capture Microscope (Arcturus Engineering) as described [25]. For each slide, at least two separate caps, one with Gal-3-positive tumour cells and one with matching, peri-tumoural (Gal-3-negative) follicular cells were obtained. DNA extracted from microdissected cells was amplified by PCR in the presence of specific primers flanking the sequence of the microsatellite markers D7S2468 and D7S6440. The first marker is located at 7q32-34 (cytogenetic localization 150.3 cM), in the same region where the human *HIPK2* gene has been mapped, and has been chosen according to the Radiation Hybrid, Genebrifge4, Stanford G3 and Genethon indications and to maximal heterozygosity [43]. The second marker is a newly identified microsatellite internal to the *HIPK2* gene (Accession Number # AY563634). This marker, that we named D7S6440, consists of a di-nucleotide repeat (CA) and its sequence variability in the general population has been recently calculated (Sciacchitano, unpublished results). All the oligonucleotide primers used in the study were synthesized and purchased from MWG Biotech and their sequences are available upon request. LOH analysis was conducted as previously described [25]. Reproducibility of each LOH was confirmed by at least two independent experiments. The FAL index was calculated as previously reported [25].

### Cell culture and plasmid constructs

The human PTC-derived K1 cells were purchased from ECACC (Salisbury, United Kingdom) and grown in DMEM:Ham's F12 (Lonza Walkersville, Inc. Walkersville, MD):MCDB-105 (Sigma Saint Louis, Missouri) (2∶1∶1) medium added with 10% FBS, and supplemented with 2 mM glutamine and 100 U/ml penicillin and streptomycin mix (Lonza).

RNAi of HIPK2 expression was obtained in K1 cells by stable transfection of pSUPERretro and pSUPER-HIPK2 constructs [10].

Overexpression of the wild type and mutant HIPK2^K221R^ was obtained by transient tranfection of K1 cells using the following constructs: pCMV-FLAG, pCMV-FLAG-HIPK2, pEGFP-HIPK2 and pEGFP-HIPK2 K221R [10]. It should be noted that K1 are a suitable and representative WDTC cellular model. They, in fact, are characterized by overexpression of Gal-3 and very low, even if detectable, levels of HIPK2.

Transfections were performed using Lipofectamine (Invitrogen) according to manufacturer's instructions. Selection of stably transfected cells was performed by treatment with puromicin at the concentration of 2 µg/ml (Sigma).

### Western Blot analysis

Total cell extracts (TCEs) were obtained as previously described [41]. Aliquots of TCEs (30–70 µg) were separated through 5%–10% SDS-PAGE and blotted onto nitrocellulose membrane (BIO-RAD). The following antibodies were used in immunoblotting: rabbit anti-HIPK2 antiserum (kindly provided by L. Schmitz), purified rat mAb anti-Gal-3 antibody (Mabtech AB, Nacka Strand, Sweden), mouse mAb anti-p53 (Santa Cruz), mouse mAb anti-p53Ser46 (Cell Signaling Technology) and mouse mAb anti-β-actin (Sigma), and HRP-conjugated anti-rabbit, anti-rat or anti-mouse, antibodies (Sigma). Immunoreactivity was detected using ECL kit (Amersham Corporation). Densitometric analysis of the intensity of the bands was performed using the software NIH ImageJ (version 1.32j).
